# Potential Child Abuse Screening in Emergency Department; a Diagnostic Accuracy Study

**Published:** 2017-01-08

**Authors:** Hossein Dinpanah, Abazar Akbarzadeh Pasha

**Affiliations:** 1 Emergency Department, Shahid Beheshti Hospital, Babol University of Medical Sciences, Babol, Iran.; 2 Urology Department, Shahid Beheshti Hospital, Babol University of Medical Sciences, Babol, Iran.

**Keywords:** Child abuse, diagnosis, emergency service, hospital, risk assessment, decision support techniques

## Abstract

**Introduction:**

Designing a tool that can differentiate those at risk of child abuse with great diagnostic accuracy is of great interest. The present study was designed to evaluate the diagnostic accuracy of Escape instrument in triage of at risk cases of child abuse presenting to emergency department (ED).

**Method:**

The present diagnostic accuracy study performed on 6120 of the children under 16 years old presented to ED during 3 years, using convenience sampling. Confirmation by the child abuse team (pediatrician, a social worker, and a forensic physician) was considered as the gold standard. Screening performance characteristics of Escape were calculated using STATA 21.

**Results:**

6120 children with the mean age of 2.19 ± 1.12 years were screened (52.7% girls). 137 children were suspected victims of child abuse. Based on child abuse team opinion, 35 (0.5%) children were confirmed victims of child abuse. Sensitivity, specificity, positive and negative likelihood ratio and positive and negative predictive values of this test with 95% CI were 100 (87.6 – 100), 98.3 (97.9 – 98.6), 25.5 (18.6 – 33.8), 100 (99.9 – 100), 0.34 (0.25 – 0.46), and 0 (0 – NAN), respectively. Area under the ROC curve was 99.2 (98.9 – 99.4).

**Conclusion:**

It seems that Escape is a suitable screening instrument for detection of at risk cases of child abuse presenting to ED. Based on the results of the present study, the accuracy of this screening tool is 99.2%, which is in the excellent range.

## Introduction

Non-accidental physical, mental, emotional or sexual abuse, or neglect of children under 18 years of age, which endangers the child’s health, comfort, and education, is defined as child abuse (1). Regardless of the culture and beliefs of a society, mistreating children may be a major health problem that requires attention from the governments and health care systems due to its wide range of long term effects. It may seem like a personal problem at first sight, however considering its probable side effects such as depression, borderline personality disorder, multiple personality disorder, attention deficit disorder, drug and alcohol abuse, prostitution, running away from home, antisocial and criminal behavior, and sexual crimes, it is considered a social and multidimensional phenomenon (2). Child abuse was first assessed as a problem that may affect the present and future life of a person in 1962 with publication of an article titled “the beaten child syndrome”, which became a stepping-stone for future studies (3). According to statistics, during 1976 to 1983 more than 50000 children were killed by their parents as a result of child abuse in the United States, and more than 25 million children were subject to abuse and anger (4). In 1995, more than 3 million children were referred to child support centers in the United States due to abuse and neglect. Death or sickness of a family member, financial problems and dissatisfaction with marriage, have been introduced as child abuse risk factors (5). In Iran, most cases of child abuse belong to physical abuse of boys and factors such as parents’ low educational level, low economic status, populated family, and mental and physical illnesses are identified risk factors (6, 7). Potential child abuse screening in those presenting to emergency department (ED) can help identify effective factors in abuse incidence and move toward reducing its prevalence by proper intervention. Designing a tool that can differentiate those at risk of child abuse with great diagnostic accuracy is of great interest for emergency physicians. Although in recent years, child abuse screening tools have significantly helped emergency physicians, the accuracy of these tools is still a matter of question (8-11). Therefore, the present study was designed to evaluate the diagnostic accuracy of Escape instrument in triage of at risk cases of child abuse presenting to ED.

## Methods

***Study design***

The present study is a prospective diagnostic accuracy study performed on children presented to ED of Shahid Beheshti and Amir Kola Hospitals, Babol, Mazandaran, Iran, during 2011 to 2014. The aim of this study was evaluating the accuracy of Escape tool in screening children at risk of child abuse. The study was approved by the ethics committee of Babol University of Medical Sciences. The researchers adhered to the principles of Helsinki Declaration and keeping patient information confidential at all stages during the study. The patients or their relatives were assured that their personal data will be confidential and only used for the purpose of the study and written informed consent was obtained from them.

***Participants***

6120 of the children (under 16 years old) presented to ED during the study period were triaged and enrolled using convenience sampling. Inclusion criteria were consent for participation, cooperation in filling out the questionnaire, and stable clinical and hemodynamic status. Cases of suicide injury, poisoning, and those who had introduced their case as child abuse or were injured by their peers were excluded. 

***Data gathering***

On admission to ED, demographic data of all children (age, sex, place of living) as well as their hydration status were recorded and Escape questionnaire for potential child abuse screening (appendix 1) was filled for them by asking questions from the child or the guardians (11). Triage was done by trained nurses. In cases of one or more abnormal answer to the questions, the screening result was considered positive. 

After admission to ED, standard treatment (based on the reason for admission) was initiated and a trained emergency medicine specialist, blind to results of screening, accurately examined the child and recorded the history regarding child abuse. In cases that were diagnosed as child abuse, the child was re-examined by the hospital’s child abuse team including a pediatrician, a social worker, and a forensic physician to confirm diagnosis. Emergency physician and all members of child abuse team were blind to the results of screening. Confirmation by the mentioned team was considered as the gold standard for identifying the patient as a victim of child abuse.

2 emergency medicine specialists passed three 2-hour educational courses with the hospital child abuse team and were responsible for initial evaluation of the patients on admission. In-charge triage nurses in this study also underwent training for a few sessions to learn about filling the questionnaire. The validity and reliability of the questionnaire were confirmed in a previous study (11).

Statistical analysis

Minimum sample size required for the present study was calculated to be 2696 cases, considering 2.3% prevalence of child abuse (11), 80% sensitivity, 95% confidence interval (CI), desired precision (d = 0.1). Data were analyzed using STATA 11.0. Quantitative variables were reported as mean and standard deviation (SD) and qualitative ones were shown as frequency and percentage. To calculate the accuracy of the tool, sensitivity, specificity, positive and negative likelihood ratios, and positive and negative predictive values and area under the receiver operating characteristic (ROC) curve were calculated with 95% CI.

**Appendix 1:** Escape questionnaire for screening child abuse

1. Is the history consistent?	Yes	No
2. Was seeking medical help unnecessarily delayed?	Yes	No
3. Does the onset of the injury fit with the development level of the child?	Yes	No
4. Is the behavior of the child, his or her care givers and their interaction appropriate?	Yes	No
5. Are findings of the head-to- toe examination in accordance with the history?	Yes	No
6. Are there signals that make you doubt the safety of the child or other family members? *if Yes describe the signals in the box Other comments below.	Yes	No
Other comments		

**Figure 1:**
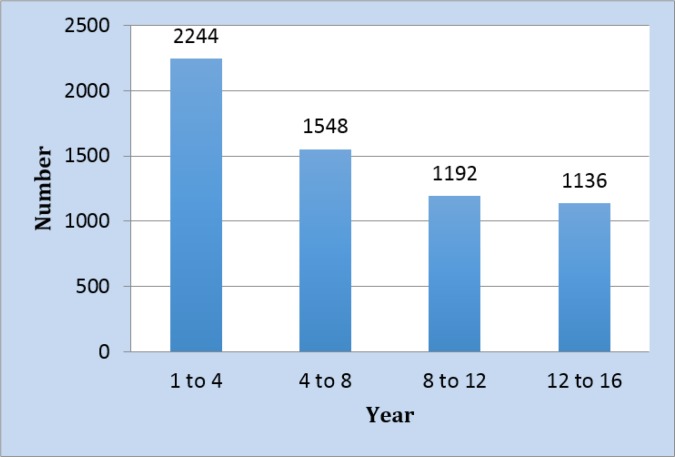
Age distribution of the studied children*.*

**Table 1:** Screening performance characteristics of the child abuse questionnaire with 95% confidence interval in prediction of at risk children presented to emergency department

**Question**	**Number (%)**	**Sensitivity**	**Specificity **	**PPV **	**NPV **
1	14 (0.2)	11.4 (0.03 – 0.27)	99.8 (99.7 – 99.9)	28.5 (0.09- 0.57)	99.5 (99.3 – 99.6)
2	26 (0.4)	20 (9- 40)	99.7 (99.5 – 99.8)	26.9 (12.4 – 48.0)	99.5 (99.3 – 99.6)
3	38 (0.6)	14.2 (5.3 -31.0)	99.4 (99.2 – 99.6)	13.1 (4.9 – 28.8)	99.5 (99.2 – 99.6)
4	27 (0.4)	17.1 (7.1 – 34.2)	99.6 (99.4 – 99.7)	22.2 (9.3 – 42.7)	99.5 (99.3 – 99.6)
5	12 (0.2)	11.4 (3.7 -27.6)	99.8 (99.7 – 99.9)	33.3 (11.2 – 64.5)	99.4 (99.2 – 99.6)
6	18 (0.3)	14.2 (5.3 – 31.0)	99.7 (99.6 – 99.8)	27.7 (10.7 – 53.5)	99.5 (99.2 – 99.6)

PPV: positive predictive value; NPV: negative predictive value.

**Figure 2:**
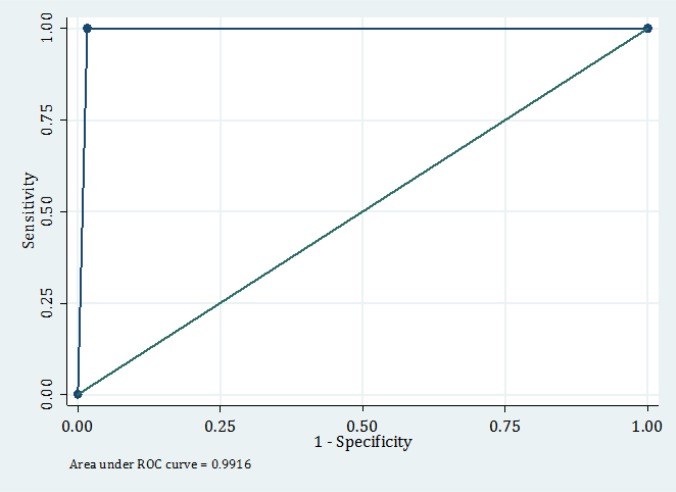
Area under the ROC curve of the child abuse questionnaire.

## Results

6120 children with the mean age of 2.19 ± 1.12 years were screened regarding potential child abuse (52.7% girls). Figure 1 shows the frequency of patients in each age group. 4376 (71.5%) of the participants resided in cities. Table 1 shows the frequency of positive answer to each of the 6 questions as well as their screening performance characteristics. Based on the results obtained from the screening questionnaire, 137 children were suspected victims of child abuse, 120 (2%) of which had 1 positive answer, 4 (0.1%) had 2 positive answers, 1 (0.01%) had 3 positive answers, and 1 (0.01%) had 4 positive answers. Finally, based on child abuse team opinion (the gold standard), 35 (0.5%) children were victims of child abuse. Sensitivity, specificity, positive and negative likelihood ratio and positive and negative predictive values of this test with 95% CI were 100 (87.6 – 100), 98.3 (97.9 – 98.6), 25.5 (18.6 – 33.8), 100 (99.9 – 100), 0.34 (0.25 – 0.46), and 0 (0 – NAN), respectively. Figure 2 shows the area under the ROC curve for the studied instrument. Area under the ROC curve was 99.2 (98.9 – 99.4).

## Discussion

Based on the findings of this study, Escape screening instrument has high sensitivity and specificity in identifying potential child abuse cases presented to ED. Area under the ROC curve of 99.2 indicates the high accuracy of the test in this regard.

As mentioned before, child abuse is a mental and health problem in every society, which is directly related to mental and physical health of the next generation. Based on the statistics reported by world health organization (WHO) about 3 million children are maltreated around the world each year and 31000 cases of murder have been reported in children under 15 years old in 2002 alone (12). Since a large number of children with various injuries are presented to ED daily, timely identification and evaluation of those suffering from or at risk of child abuse plays a significant role in preventing further damages. Child abuse rate reported in various studies carried out in EDs has ranged from 2% to 10% (13-19). Using a standard tool that can accurately determine true cases is a challenge for physicians in supporting children’s rights. Protocols designed for this purpose should be able to guide the physicians toward a comprehensive answer with few questions. In 2012, Louwers et al. used Escape screening instrument in 3 health centers for the first time.

In that study, Escape was used to evaluate potential risk of child abuse in children (aged 0 to 18 years) presented to the EDs. Using this instrument, screening rate increased from 20% in February 2008 to 67% in December 2009. Detection rate in the screened children was 5 times higher than those not screened. Therefore, it seems that Escape tool is effective in increasing detection of potential child abuse (20). 

Pless et al. studied the Accident- Suspected child abuse and neglect (A-SCAN) method, a checklist with 10 questions for assessing the risk of child abuse. The results of this checklist correlated with physical examination results reported by the physician. No significant increase in detection of child abuse was seen after introduction of this method. This could mean that ED staff were already doing well and the method used was not efficient (19). In another study to assess child abuse by consulting the child protection register, a flowchart with 4 questions was included in the patient’s file. Results showed that inclusion of a flowchart improved awareness, attention and documentation of suspected abuse cases (15). In Bleeker et al. study, a 9-question checklist was used in ED for collecting information from children suspected to be child abuse cases, which using this tool the number of detected cases increased (21). 

Hosseinkhani and colleagues determined the status of child abuse in the Iranian population and evaluated the validity and reliability of a new questionnaire. They concluded that, their questionnaire is a new tool with acceptable validity and reliability and can be applied in child abuse studies in Iran (22).

Since currently there is no accepted standard for screening children at risk of child abuse in ED, researchers are trying to design and develop new decision rules or validate the existing tools. Therefore, the present study was designed with the same aim. It seems that carrying out similar studies in other parts of the country with various cultural and economic statuses can provide more acceptable results for reaching a decision regarding the accuracy of this tool.

## Conclusion:

It seems that Escape is a suitable screening instrument for detection of at risk cases of child abuse presenting to ED. Based on the results of the present study, the accuracy of this screening tool is 99.2%, which is in the excellent range.
